# Normalization of blood clotting characteristics using prothrombin complex concentrate, fibrinogen and FXIII in an albumin based fluid: experimental studies in thromboelastometry

**DOI:** 10.1186/s13049-021-00867-5

**Published:** 2021-04-09

**Authors:** Tobias Koller, Nadia Kinast, Andres Guilarte Castellanos, Sergio Perez Garcia, Pilar Paniagua Iglesias, Xavi León Vintro, Jose Mateo Arranz, Noelia Vilalta Seto, Ma. Victòria Moral García, Ana Belén Moreno-Castaño, Jose Aznar-Salatti, Gines Escolar Albaladejo, Maribel Diaz-Ricart

**Affiliations:** 1 Hospital de la Santa Creu i San Pau, Universidad Autonoma de Barcelona, Departamento de Cirugía, Carrer de Sant Quintí, 89, 08041 Barcelona, Spain; 2 Consorci Sanitari Alt Penedés-Garraf, Carrer de l’Espirall, s/n, Vilafranca del Penedés, Spain; 3 Hospital Mútua Terrassa, Plaça del Doctor Robert 5, 08221 Terrassa, Barcelona Spain; 4 Hematopathology, Pathology Department, CDB, Hospital Clinic, IDIBAPS, University of Barcelona, Barcelona, Spain; 5 CSL Behring S.A, Barcelona, Spain

**Keywords:** Massive bleeding, Blood transfusion, Fibrinogen, Factor XIII, Prothrombin complex concentrate, Fluid therapy, Hemostatic resuscitation, Plasma substitutes

## Abstract

**Background:**

Colloid fluids supplemented with adequate combinations of coagulation factor concentrates with the capability to restore coagulation could be a desirable future treatment component in massive transfusion.

**Methods:**

Starting from a coagulation factor and blood cell-free albumin solution we added Prothrombin Complex Concentrate, Fibrinogen Concentrate and Factor XIII in different combinations and concentrations to analyze their properties to restore thromboelastometry parameters without the use of plasma. Further analysis under the presence of platelets was performed for comparability to whole blood conditions.

**Results:**

Albumin solutions enriched with Fibrinogen Concentrate, Factor XIII and Prothrombin Complex Concentrate at optimized concentrations show restoring coagulation potential. Prothrombin Complex Concentrate showed sufficient thrombin formation for inducing fibrinogen polymerization. The combination of Prothrombin Complex Concentrate and Fibrinogen Concentrate led to the formation of a stable in vitro fibrin clot. Fibrinogen and Factor XIII showed excellent capacity to improve fibrin clot firmness expressed as Amplitude at 10 min and Maximal Clot Firmness. Fibrinogen alone, or in combination with Factor XIII, was able to restore normal Amplitude at 10 min and Maximal Clot Firmness values. In the presence of platelets, the thromboelastometry surrogate parameter for thrombin generation (Clotting Time) improves and normalizes when compared to whole blood.

**Conclusions:**

Combinations of coagulation factor concentrates suspended in albumin solutions can restore thromboelastometry parameters in the absence of plasma. This kind of artificial colloid fluids with coagulation-restoring characteristics might offer new treatment alternatives for massive transfusion.

**Trial registration:**

Study registered at the institutional ethic committee “Institut de Recerca, Hospital Santa Creu i Sant Pau, with protocol number IIBSP-CFC-2013-165.

## Background

Massive bleeding can cause severe intravascular hypovolemia with significant hypoperfusion of peripheral tissues leading to life-threatening hemorrhagic shock. The correction of the underlying hypovolemic state with conventional resuscitation fluids like crystalloid or colloid solutions contributes to bleeding associated coagulopathies by dilution of plasma coagulation factors [1]. Deterioration of physical clot strength and, in a later stage of prolonged bleeding, reduced thrombin generation potential are the principal coagulopathic patterns found when monitored by viscoelastic testing [2]. Viscoelastic testing, like rotational thromboelastometry (TEM), is frequently used as a point-of-care tool in severe bleeding, providing comprehensive information about the viscoelastic and temporal characteristics of blood clot formation [3]. Thereby identified coagulopathies caused by single- or combined coagulation factor deficits are increasingly treated with coagulation factor concentrates (CFC), like Fibrinogen Concentrate (FC), Prothrombin Complex Concentrate (PCC) and Factor XIII Concentrate (FXIIIC) [4–6]. The principle goal of CFC-based treatment strategies is to reduce transfusion rates of allogeneic blood products and adverse events associated with plasma transfusion [7]. There is growing scientific evidence that coagulation factor deficiencies in bleeding patients can be effectively and safely treated with CFCs with some studies showing better outcomes for CFCs when compared to plasma transfusion [8–10]. By contrast there is a certain paucity of high-quality evidence in favor of plasma, although plasma transfusion is still a widely accepted standard of trauma and non-trauma massive transfusion protocols [11]. The high acceptance of plasma transfusion among many physicians might be explained, not only by its stabilizing effect on the coagulation system providing a close-to-physiological factor composition but also by its resuscitation fluid quality with good intravascular volume effects in patients with hemorrhagic shock. Currently, no alternative products are available which share these two characteristics with human plasma. Therefore, colloid fluids providing adequate intravascular volume effects combined with maintained hemostatic properties could be an interesting future treatment component in massive transfusion and damage control resuscitation, helping to reduce plasma transfusion and its associated side effects under maintenance of the stabilizing effects on coagulation and hemodynamics.

We hypothesized, that an optimized and well-balanced combination of different coagulation factors, reconstituted in an albumin-based carrier solution, would provide basic clotting characteristics with TEM responses compatible with whole blood if tested under the presence of platelets.

In this study, we used a thromboelastometric approach to analyze modifications of viscoelastic parameters in a plasma-, and blood cell-free environment. It is technically feasible to perform thromboelastometric analysis in such conditions, although this approach has been limited to its use in laboratory investigations [12, 13].

## Methods

### Experimental design

This study (protocol number IIBSP-CFC-2013-165) was designed to explore in vitro the capability of CFCs to restore coagulation properties. A series of experimental studies were performed to define the optimal factor concentrations of such coagulation resuscitating fluid (CRF). Starting from a coagulation factor and blood cell-free solution of 5% human albumin we added PCC, FC and FXIIIC in different combinations and concentrations to analyze their properties to restore thromboelastometric parameters without the use of plasma. The optimal CFC composition was further analyzed under the presence of platelets to improve comparability to whole blood conditions.

All coagulation factors – fibrinogen (FGN), factor XIII (FXIII) and prothrombin complex (PC) factors II, VII, IX and X - were derived from commercial CFCs (FC, FXIIIC and PCC). The optimal concentration of FGN and FXIII in CRF was determined by direct comparison to clot firmness parameters of plasma from an internal control group. The optimal concentration of PC coagulation factors was determined by the shortest obtained clotting time (CT) value. CRF was considered as having a coagulation restoring potential to allow consideration as plasma substitute, if thromboelastometric responses of the final CRF composition lay within the normal range for whole blood when tested in presence of platelets [14].

### Plasma reference values for thromboelastometry parameters

TEM parameters were determined from plasma from healthy volunteers to define the reference range for clotting time (CT), the amplitude at 10 min (A10) and maximum clot firmness (MCF) under blood cell-free conditions. For this purpose, we collected blood samples from 33 healthy volunteers who had not taken medication affecting coagulation- or platelet-function in the last 10 days. A sample of 4.5 ml of blood was drawn from each donor in citrated tubes (0.12^9^ M) and centrifuged at 3200 rpm for 25 min. Plasma supernatant was used for FIBTEM analysis. FIBTEM was used to minimize viscoelastic signals associated with residual platelets after centrifugation. The 95% confidence interval of the obtained values was defined as the reference range for plasma. The established reference ranges were later used for comparison with TEM responses of CRF samples to determine the optimized CRF factor concentration.

### Study samples

#### Composition of study samples

Study samples were composed of an artificial fluid solution (AFS) based on 5% human albumin together with different CFC combinations and concentrations. 20% human Albumin (Grifols®, Spain) was diluted with an isotonic, balanced, crystalloid solution (Viaflo Plasmalyte® 148, Baxter, Spain) to a final albumin concentration of 5% correspondent to high physiologic plasma concentrations for albumin. Calcium gluconate was added to achieve a physiological free ionized calcium concentration of 1.0–1.2 mmol/l. The solution was buffered with TRIS buffer (1 M) to a physiological pH range between 7.36–7.45. Electrolyte concentrations and pH were measured on the blood gas analyzer Radiometer®ABL 90 Flex to confirm the physiological composition of our stem solution.

All factor concentrates were provided by CSL Behring. The different coagulation factor concentrates (CFC) were reconstituted in stock solutions. PCC (Beriplex, CSL Behring GmbH, Germany), FC (Riastap, CSL Behring GmbH, Germany) and FXIIIC (Fibrogammin/Cluvot, CSL Behring GmbH, Germany) were used as CFCs for this in vitro analysis. The lyophilized proteins were reconstituted with the minimum amount of the accompanied provider’s solution necessary for protein dissolving, resulting in final concentrations of 0.025 IU/μl for factor IX (FIX) as reference protein in PCC, 0.1 mg/μl for FGN, and 0.05 IU/μl for FXIII. High final protein concentrations in the stock solutions were necessary to avoid dilutional effects during the preparation of the final study samples. The stock solution was directly used or stored at − 70 °C for later use. The final composition of AFS free of proteins and blood cells was tested as a negative control with EXTEM and FIBTEM subtests as described in the thromboelastometry section.

#### Preparation of study samples

Aliquots of the stock solution containing coagulation factors were added to the AFS within citrated tubes to reach the defined final factor concentration. The study samples were warmed to 37 °C in the provided warming chamber of the ROTEM® machine before testing.

### Variable CFC concentrations and platelet count

The effect of various combinations of CFC-derived coagulation factors suspended in AFS on their functional contribution to clot formation was evaluated by TEM. The study samples were distributed in different test series. Within the same test series the protein concentration of only one component (FGN, FXIII or PC), or platelet count, was gradually modified while the concentration of the other components was left unchanged. The protein concentration of PC used in our in vitro-experiments is provided as IU/ml referring to the underlying factor IX activity, being the PC reference protein. Three main series of tests were performed combining different CFC-derived proteins added to the AFS:

a) Increasing PC concentrations (0.05, 0.1, 0.25, 0.5, 1, 2 and 4 IU/ml) over a fixed FGN concentration of 4 g/l; b) Increasing FGN concentrations (0.5, 1, 2, 4, 8 and 12 g/l) over a fixed PC concentration of 1 IU/ml; c) Increasing FXIII concentrations (0.1, 0.5, 1, 2, 4 and 8 IU/ml) over a fixed PC concentration of 1 IU/ml and a fixed FGN concentration of 4 g/l. Each concentration step of the changing factor component defined one study sample that was tested by TEM. The study samples were analyzed for viscoelastic properties with the FIBTEM-S subtest. A TEM response within the defined reference values (Table 1) for A10 and MCF derived from internal controls determined the optimal FGN and FXIII composition of the CRF. Combined FGN/FXIII preparations with TEM responses within the normal range for A10/MCF were given priority to single factor preparations (FGN alone) for defining the final CRF composition. The shortest CT value obtained in a series with increasing PC concentrations defined the final PC concentration of CRF.

**Table 1:** Reference ranges for standard TEM parameters CT, A10, MCF. Reference ranges are presented for plasma and whole blood. Plasma ranges are derived from an internal control group. Values from an external control group from previous studies are also highlighted for comparison. Whole blood reference ranges are shown for comparison with TEM results of CRF under presence of platelets. The recommended treatment thresholds are presented to provide a clinical context of the obtained TEM results

Reference ranges	Plasma (Internal control)^**a**^	Plasma (External control)^**b**^	Whole blood^**c**^	Treatment threshold^**d**^
**CT (*sec*)**	47–54	53–71	42–74	> 80
**A10 (*mm*)**	17–24	n.a.	43–65	< 7
**MCF (*mm*)**	18–26	17–35	49–71	< 14

^a^ Values derived from fresh plasma from 33 healthy, non-medicated volunteers. Reference range corresponds to 95% confidence interval

^b^ Values derived from fresh plasma as published by Schörgenhofer et al. [13]

^c^ Values derived from whole blood as published by Lang et al. [14]

^d^ Treatment thresholds for whole blood EXTEM-CT and whole blood FIBTEM-A10/MCF as published by

Schöchl and Schlimp [15]. and Ranucci et al. [16]

In a fourth test series the effect of platelets on samples containing the final CRF composition of PC (1 IU/ml), FGN (4 g/l) and FXIII (1 IU/ml) was evaluated adding an increasing number of washed platelets (12.5, 25, 50, 100, 200 and 400 platelets 10^3^/μl) obtained from healthy, non-medicated donors. The samples were analyzed with the FIBTEM-S and EXTEM-S subtests.

### Preparation of platelet suspensions

Blood was collected into citrate/phosphate/dextrose (final concentration of citrate of 19 mM) and centrifuged (120 x g for 15 min) to obtain platelet-rich plasma. Washed platelets were obtained by mixing PRP with equal volumes of citrate/acid citric/dextrose (93 mM sodium citrate, 7 mM acid citric, and 140 mM dextrose), pH 6.5 containing 5 mM adenosine and 3 mM theophylline (CCD–AT) [17]. The final pellet was resuspended in a Hanks’ balanced salt solution (136.8 mM NaCl, 5.3 mM KCl, 0.6 mM Na2HPO4, 0.4 mM KH2PO4, 0.2 mM NaH2PO4-2H2O) supplemented with dextrose (2.7 mM) and NaHCO3 (4.1 mM), pH 7.2, and maintained for 50 min at 37 °C before experiments were performed. Concentrated washed platelets were added to the study samples to reach the established platelet counts.

### Thromboelastometry

TEM analysis was performed on the ROTEM® delta machine (Rotation Thromboelastometry, TEM International, Munich, Germany). Plastic cups were filled with 300 μl of pre-warmed (37 °C) solutions. FIBTEM-S/EXTEM-S subtests were used providing extrinsic coagulation activation with/without cytochalasin-based deactivation of platelets. FIBTEM is essentially dependent on the FGN function. This test inhibits the platelet contribution to clot formation, leaving only the clotting proteins. Thus, one can observe the contribution of functional FGN to clot formation. A minimum of three independent measurements on different, freshly prepared samples were performed for each concentration step. Thromboelastometry measurements were performed immediately after combining the different protein and/or cellular components of the study samples. Standard TEM parameters were obtained for statistical analysis: CT, A 10, and MCF.

### Statistical analysis

The 95% confidential interval defined the normal range for the obtained TEM parameters of the internal control group: CT, A10, and MCF. Statistical analysis was performed on SAS® 9.3 Statistical Software. The correlation analysis of the collected data was performed on basis of a dispersion graph for illustrating values of TEM parameters in function of the corresponding dose. Linear correlation was analyzed on basis of the underlying dispersion graph. In the case of linear correlation the Pearson coefficient for linear correlation was applied. For interpreting our data *p* < 0.050.05 were considered significant.

## Results

### Negative control of coagulation factor free AFS samples

No response was detected in FIBTEM or EXTEM subtests when performed on AFS that were not enriched with coagulation factors. The tests resulted in an infinite CT value and no clot formation could be detected.

### Normal range of plasma tested by TEM

The 95% confidence interval of the TEM parameters determined in 33 plasma samples of healthy volunteers was defined as the “normal range” for plasma. The results are summarized in Table 1 as “Internal Control”. The upper limit of normal for CT in our internal control group was 53 s for EXTEM subtests and 54 s for FIBTEM subtests. The obtained plasma reference range for A 10 and MCF was 17–24 mm and 18–26 mm, respectively. Previously published reference ranges for standard TEM parameters for plasma and whole blood and usually accepted treatment thresholds are shortly summarized in Table 1 for comparison [13–16].

### Combination of PC and FGN leads to fibrin clot formation in a plasma and platelet free environment

**Fig. 1:**
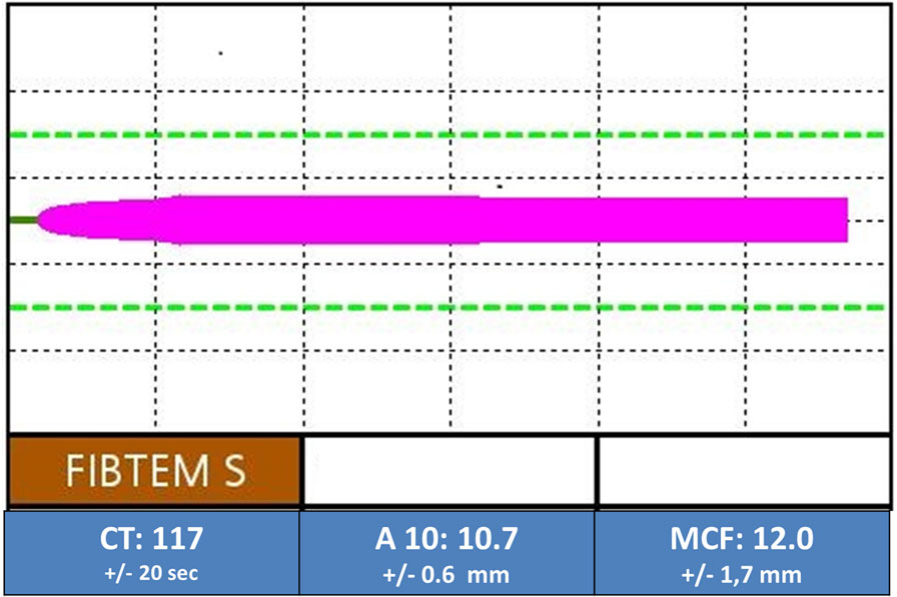
Combination of PCC and FGN leads to fibrin clot formation in a plasma and platelet free environment. Figure is representative for fibrin clot formation under a combination of PCC at 1 IU/ml with FGN at 4 g/l.

The combination of PC and FGN in an artificial colloid solution free from other blood components leads to the formation of a thromboelastometrically measurable fibrin clot. The formed fibrin clots were stable as no significant deterioration of its viscoelastic integrity was observed during the 60 min TEM response. Figure 1 shows a typical TEM graph using a FGN concentration of 4 g/l and PC at 1 IU/ml (using the concentration of FIX as a reference). The viscoelastic properties of the tested fluids at this coagulation factor combination (CT 117 ± 20 s, A10 10.7 ± 0.6 mm, MCF 12 ± 1.7 mm) were located outside the aspired, predefined normal values (Table 1), especially CT was considerably prolonged. Fibrin formation was dependent on the presence of PC as negative controls without PC proteins ruled out spontaneous fibrin formation during TEM analysis.

### Increasing concentrations of PC caused progressive improvements in TEM parameters at a fixed FGN concentration of 4 g/l

As described in Table 2, a progressive shortening of CT was observed with increasing concentrations of PC. An inverse association between PC concentration and CT was observed within the 0.05–1 IU/ml concentration range with the shortest CT detected at a concentration of 1 IU/ml and an average CT of 117 s. This value was significantly prolonged when compared to the CT reference range of our internal control group, to external controls, and published treatment thresholds (Table 1). Higher PC concentrations (> 1 IU/ml) did not further shorten CT values. Even in very high concentrations like 4 IU/ml, CT did not further improve.

**Table 2:** Analysis of viscoelastic parameters in albumin-based colloid solutions enriched with prothrombin complex concentrate (PCC) and fibrinogen (FGN). Different PCC concentrations (0.05, 0.1, 0.25, 0.5, 1, 2, 4 IU/ml) were combined with fixed fibrinogen (FGN) concentrations of 4 g/l. The albumin concentration was maintained stable at 5%. Negative controls on PCC-free solutions did not show any measurable ROTEM response (infinite CT). A minimum of three repeats for each concentration step was performed. The value in bold letters is prolonged (>80s) but still reflects the optimal PCC response for CRF composition

***Increasing PCC concentrations over FGN 4 g/l***							
PCC conc.^a^	0,05	0,1	0,25	0,5	1	2	4
CT^b,*^	613 ± 251	337 ± 46	216 ± 50	168 ± 22	**117 ± 20**	95 ± 40	115 ± 18
A10^c,**^	5.7 ± 1.5	6.3 ± 1.5	6.7 ± 0.6	8.3 ± 1.5	10.7 ± 0.6	14 ± 4.3	11.7 ± 2.1
MCF^c,***^	6.3 ± 1.5	7.3 ± 2.1	7 ± 1	9.3 ± 2.3	12 ± 1.7	14.3 ± 4.9	12 ± 2.6

*p < 0,0001, Pearson rho 0,9 for1/CT, **p = 0,0013, Pearson rho 0,65, ***p = 0,0063, Pearson rho 0,57

^a^PCC concentrations in IU/ml refer to final factor IX concentrations as reference protein in this product

^b^CT in seconds. Mean values ± SD

^c^A10 and MCF in mm. Mean values ± SD

Effects of increasing concentrations of PC on fibrin clot strength measured by A 10 and MCF were less evident than those observed on CT. As shown in Table 2, a positive linear correlation between rising PC concentrations and thromboelastometry clot strength parameters was seen (Pearson correlation coefficient of rho = 0.65, 0.57 and *p*-values < 0.0013, 0.0063 for A10 and MCF respectively).

According to these data, a concentration of 1 IU/ml PC for the final CRF composition was determined.

### Rising FGN concentrations improved clot strength related parameters at a fixed PC concentration of 1 IU/ml

Based on the previous results, a fixed PC concentration of 1 IU/ml was chosen for this test series as it provided the shortest CT values in our model. As summarized in Table 3, a negative non-linear correlation was seen between increasing FGN concentrations and measured CT values. This effect was more pronounced in the lower FGN range between 0.5 and 2.0 g/l and became less significant with higher FGN concentrations above 2 g/l.

**Table 3:** Analysis of viscoelastic parameters in albumin-based colloid solutions enriched with fibrinogen (FGN) and prothrombin complex concentrate (PCC). Different FGN concentrations (0.5, 1, 2, 4, 8, 12 g/l) were combined with fixed PCC concentrations of 1 IU/ml. The albumin concentration was maintained stable at 5%. Negative controls on fibrinogen free solutions did not show any measurable ROTEM response (infinite CT). A minimum of three repeats for each concentration step was performed. The values in bold letters reflect the optimal FGN response for CRF composition

***Increasing FGN concentrations over PCC 1 IU/ml***						
FGN conc. ^a^	0,5	1	2	**4**	8	12
CT ^b,*^	4032 ± 1949	620 ± 620	221 ± 90	117 ± 20	154 ± 27	198 ± 1
A10^c,**^	n.a.	2.7 ± 0.6	3.6 ± 0.6	**10.7 ± 0.6**	25.7 ± 4.2	47 ± 1.4
MCF^c,***^	n.a.	3.7 ± 0.6	3.3 ± 0.6	**12 ± 1.7**	26.3 ± 3.1	49.5 ± 3.5

*p < 0,0001, Pearson rho 0,84 for1/FGN, **p < 0,0001, Pearson rho 0,98, ***p < 0,0001, Pearson rho 0,98

^a^FGN concentrations in g/l

^b^CT in seconds. Mean values ± SD

^c^A10 and MCF in mm. Mean values ± SD

Moreover, FGN concentrations rising from 0.5 to 12 g/l improved the TEM parameters that characterize the clot strength with a strong positive correlation between FGN concentrations and the corresponding TEM parameters (Pearson correlation coefficient of rho = 0.98 and *p*-values of < 0.0001 for both A10 and MCF respectively, Table 3). At a physiological FGN concentration of 4 g/l the measured TEM responses (11/12 mm for A 10/MCF) were slightly below the desired TEM range of 17 mm. The final FGN concentration for CRF composition of 4 g/l was determined in synopsis with the results achieved in combination with FXIII.

### Increasing concentrations of FXIII (0.1, 0.5, 1, 2, 4, 8 IU/ml) enhanced clot strength at fixed concentrations of PC (1 IU/ml) and FGN (4 g/l)

Fixed concentrations of FGN (4 g/l) and PC (1 IU/ml) were used in this experimental setting. FXIII effects on fibrin clot strength were evaluated by A 10 and MCF. As shown in Table 4**,** a moderate to high positive correlation was observed for the Pearson correlation coefficient between rising FXIII concentrations and A10 and MCF (*p*-values of 0.004 and 0.002, respectively). The previously observed TEM response at a FGN concentration of 4 g/l (MCF 12 mm) in a FXIII-free environment (MCF 12 mm), significantly improved to MCF of 24 mm by adding 1 IU/ml of FXIII, thus reaching the upper limit of our predefined range for normality. No additional effect or statistical correlations were observed for other tested TEM parameters (CT). According to the combined data on FGN and FXIII, final concentrations of 4 g/l for FGN and 1 IU/ml for FXIII were determined for further analysis of CRF under the presence of platelets.

**Table 4:** Analysis of viscoelastic parameters in albumin-based colloid solutions enriched with factor XIII (FXIII), prothrombin complex concentrate (PCC) and fibrinogen (FGN). Different FXIII concentrations (0.1, 0.5, 1, 2, 4, 8 IU/ml) were combined with fixed PCC and FGN concentrations of 1 IU/ml and 4 g/l, respectively. The albumin concentration was maintained stable at 5%. Negative controls for FXIII- free solutions are represented by the corresponding compositions shown in Tables 2 and 3. A minimum of three repeats for each concentration step was performed. The values in bold letters reflect the optimal FXIII response for CRF composition

***Increasing FXIII concentrations over PCC 1 IU/ml and FGN 4 g/l***						
FXIII conc. ^a^	0.1	0.5	**1**	2	4	8
CT^b,*^	123 ± 12.7	128 ± 17.3	135.3 ± 12.7	122.3 ± 7.5	133.5 ± 29	133.3 ± 22.2
A10^c,**^	12.5 ± 0.7	16.3 ± 1.5	**21.3 ± 2.1**	24.3 ± 2.3	30.3 ± 11.4	30.7 ± 9.9
MCF^c,***^	13.5 ± 0.7	18.7 ± 2.1	**23.7 ± 3.1**	26.7 ± 4.6	32.3 ± 10.4	35.7 ± 12.4

*no correlation, **p = 0,004, Pearson rho 0,65, ***p = 0,002, Pearson rho 0,69

^a^FXIII concentrations in IU/ml

^b^CT in seconds. Mean values ± SD

^c^A10 and MCF in mm. Mean values ± SD

### Platelets completely restore whole blood TEM parameters, including CT

The impact of increasing platelet counts (12.5, 25, 50, 100, 200, 400 × 10^3^/μl) on various TEM parameters was investigated under the previously established conditions using fixed CRF concentrations of PC (1 IU/ml), FXIII (1 IU/ml) and FGN (4 g/l) as this combination provided optimal values for CT and clot strength in TEM studies.

**Fig. 2:**
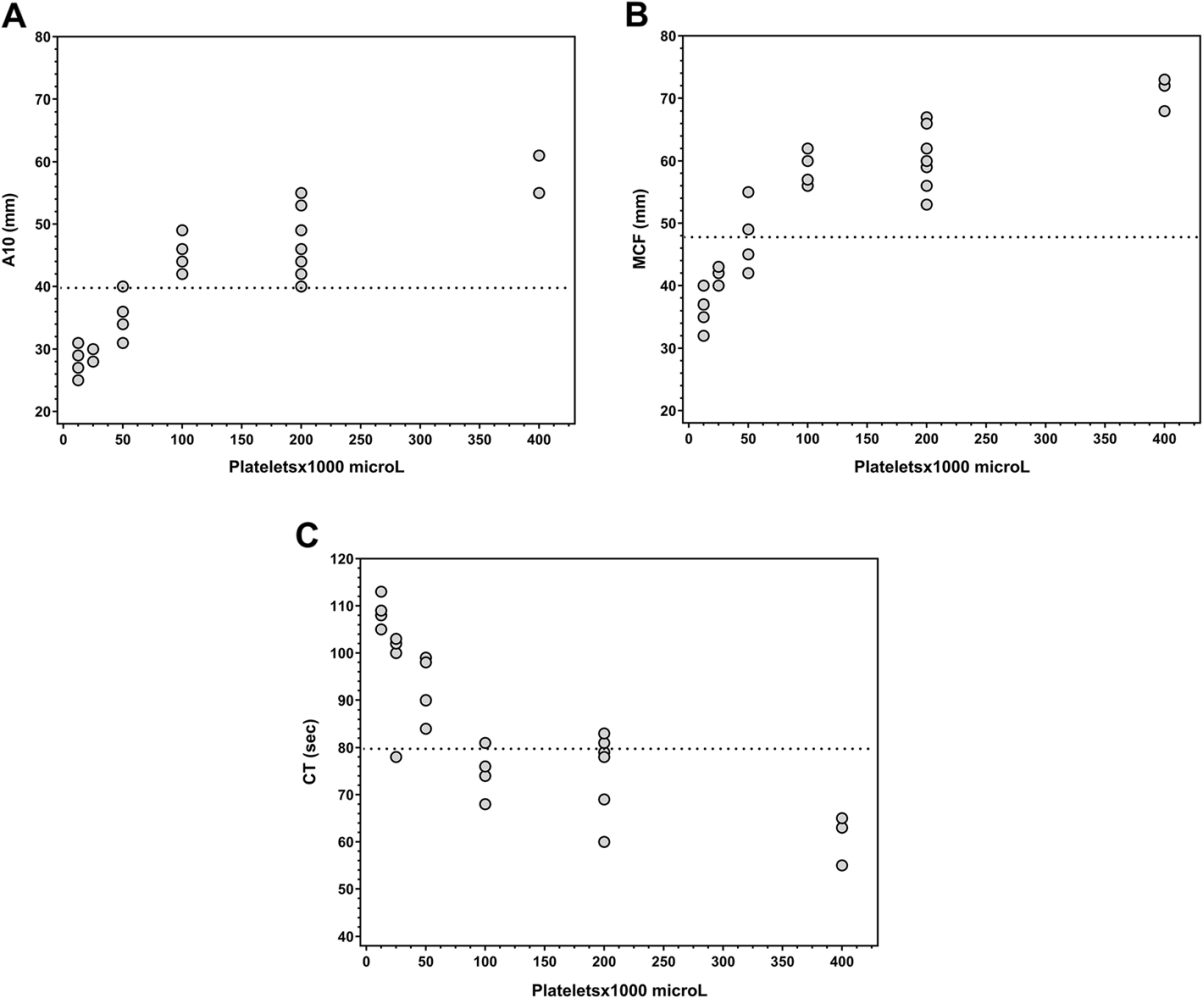
Analysis of viscoelastic parameters of CRF, defined as albumin-based colloid solution containing PCC [1 IU/ml], FXIII [1 IU/ml] and FGN [4 g/l]). Different platelet concentrations (12.5, 25, 50, 100, 200, 400 × 10^3^/μl) were combined with CRF. A minimum of three repeats for each concentration step was performed. The continuous line in the graph shows the lower limit of normal range for whole blood TEM parameters. **a** Effect of different platelet concentrations on A10. **b** Effect of different platelet concentrations on MCF. **c** Effect of different platelet concentrations on CT

The presence of platelets (in the optimally designed CRF) significantly improved fibrin clot strength parameters assessed by A10 and MCF (Fig. 2a and b)**.** A moderate to high positive correlation was observed between rising platelet concentrations and A10 and MCF measurements (Pearson correlation coefficient of rho = 0.89/0.86, respectively and *p*-values of 0.0001). Clot strength values reached the normal range for EXTEM whole blood under a minimum platelet concentration around 100 × 10^3^/μl, as calculated from a regression analysis. No effect on clot strength was observed for platelets in the FIBTEM tests performed in parallel.

As shown in Fig. 2c**,** the presence of platelets (in the optimal CRF) shortened CT. A moderate to high negative correlation was observed with rising platelet concentrations and measured CT (Pearson correlation coefficient of rho = − 0.80, *p* < 0.0001). TEM values reached normal whole blood CT values for EXTEM subtests under a minimum platelet concentration of around 100 × 10^3^/μl.

As shown in Table 5, CRF at PC 1 IU/ml, FGN 4 g/l, FXIII 1 IU/ml reached TEM parameters comparable to whole blood EXTEM reference ranges when tested under the presence of 100 × 10^3^/μl platelets.

**Table 5:** Comparison of whole blood reference ranges with TEM results for CRF under presence of 100 × 10^3^/μl platelets

	WHOLE BLOOD^**a**^	CRF + PLATELETS
**CT (s)**	42–74	74 +/− 5
**A 10 (mm)**	43–65	45 +/− 3
**MCF (mm)**	49–71	59 +/− 2

^a^ Whole blood reference ranges as published by Lang et al. [14]

## Discussion

Data from our in vitro study demonstrate that it is possible to restore coagulation properties by combining defined concentrations of coagulation factor concentrates in an albumin-based colloid solution. The viscoelastic clot formation parameters A 10 and MCF observed in CRF were comparable not only to human plasma, but also to whole blood under the presence of platelets. The surrogate parameter for thrombin generation, CT, was prolonged when compared to our internal control group, but reached normal levels for whole blood when analyzed in presence of platelets at 100 × 10^3^/μl [14]. Fluid substitutes like CRF could be an interesting treatment option, with clinical indications comparable to those of fresh frozen plasma.

Massive bleeding, independent of its etiology (trauma, obstetrical or surgical), usually includes high ratio FFP transfusion guided by institutional massive transfusion protocols, based on a large body of evidence favoring plasma against coagulation factor-free resuscitation fluids [11, 18]. This hemostatic resuscitation concept, however, is insufficient to avoid massive transfusion-associated multifactor coagulopathies [19]. Factor containing fluids for volume therapy, like CRF, that are characterized by optimized viscoelastic properties could be an appealing new treatment component. Additional point-of-care monitoring would still allow for goal-directed top-up corrections, but monitoring intensity could be reduced. Easy storage of the CRF components at 4 °C, immediate availability without thawing time, and the universal applicability, independent of blood group compatibility, could provide both logistic and clinical advantages of this new product compared to frozen plasma. The clinical indications for CRF administration could largely be analog to those of plasma, focusing on uncontrolled bleeding events related to multifactor deficiencies in hypovolemic patients, in which ongoing factor-free fluid therapy could cause further deterioration of the basic coagulation mechanisms. In these urgent scenarios CRF could provide shorter decision-to-treatment times than those known for plasma transfusion. CRFs could easily be held available, independent of blood bank facilities, in all areas exposed to massive bleeding scenarios, like operating theatres, ICUs, delivery rooms, emergency departments and even in prehospital emergency- or military settings.

Future clinical studies in massive transfusion scenarios will have to show if substituting plasma by CRF is feasible and if equal efficacy in terms of hemodynamic and coagulation stability will be provided. Furthermore clinical trials will have to show if plasma-specific adverse events like “transfusion-related acute lung injury (TRALI)” or “transfusion-related immune modulation (TRIM)” would be less frequent under treatment of purified plasma-derived components like CFCs or albumin. It is not finally established from previous clinical studies, if the overall complication rate, especially when compared to solvent and detergent treated pooled plasma (S/D plasma), really results in a better safety profile for factor concentrates. Next to these still to be answered clinical issues, current prices for the final CRF components would imply a major economic obstacle for the implementation of this product into clinical routine, even if outcome superiority compared to plasma-based standard of care could be demonstrated in future clinical studies.

Human albumin 5% was chosen as carrier solution for the coagulation factor compound of our CRF for different reasons. First, colloids were favored against crystalloids, because accurately defined coagulation factor concentrations within a predefined volume of a resuscitation fluid would only make sense, if the underlying carrier showed adequate and sustained volume effects in the intravascular space. Second, the colloid should not interfere in a significant manner with the coagulation system. Although all colloid solutions show dilutional effects, albumin solutions, together with gelatins, seem to cause less colloid-specific, detrimental effects on platelet function or fibrin polymerization than other colloids, like dextrans, or starches [20, 21]. We preferred albumin against gelatins to optimize comparability to FFP in future trials. Experimental studies show that albumin-based colloid solutions provide stabilizing effects on the endothelial barrier and show intravascular plasma expander effects of nearly 100% [22, 23]. By contrast, no such effects on the endothelial barrier could be demonstrated for CFCs [24]. The administration of well-balanced coagulation factors in carrier solutions with constant intravascular volume effects might be a safe way to treat bleeding associated coagulopathies, as “overshot” peak plasma concentrations caused by the infusion of highly concentrated factor formulas (as under non-diluted CFC administration) would be avoided. The intravascular volume effect of colloidal resuscitation fluids seems to be context-sensitive and correlated to the integrity of the endothelial glycocalyx layer. There is growing evidence that in special clinical conditions like sepsis and trauma, characterized by elevated glycocalyx shedding rates, the volume expander rate of isooncotic colloids would be less than predicted. The underlying glycocalyx disruption seems to be partially driven by a “low-protein environment” caused by aggressive crystalloid or synthetical colloid fluid treatment. By contrast, protein containing resuscitation fluids like plasma or albumin-based colloids seem to provide protective effects against glycocalyx shedding. This albumin-mediated protection of the glycocalyx layer is currently demonstrated in mostly preclinical, in-vitro studies, and it remains a matter to future studies if this translates into a clinically detectable advantage of albumin containing resuscitation fluids [25, 26] .

Hemostasis is a result of coordinated interactions between platelets and coagulation mechanisms [27]. Coagulation mechanisms necessary for consolidation of platelet mediated primary hemostasis require a cascade of enzymatic reactions leading to the formation of fibrin [15, 28, 29]. Results of the present study indicate that coagulation mechanisms can be reproduced using a restricted number of coagulation factors suspended in a neutral fluid. To our knowledge, this is the first experimental study that has been able to demonstrate that the combination of commercially available CFCs in an initial coagulation factor- and blood-cell-free solution leads to the formation of a stable in vitro fibrin clot. The initiation of the coagulation in this fluid requires the use of EXTEM or FIBTEM reagents whose components (calcium, phospholipids and tissue factor) would trigger the activation of the prothrombin complex coagulation factors VII, IX, X and II contained in commercial PCCs [6]. These coagulation factors lead to sufficient thrombin generation and warrant the basic activating mechanism of the coagulation system to sustain in vitro fibrin polymerization. FGN provides the structural clotting substrate supporting secondary hemostasis [30]. The necessary FGN concentration in CRF to reach normal TEM values (when combined with FXIII) was found in the range of physiological plasma concentrations, around 4 g/l for FGN and 0,5–1 IU/ml for FXIII. FXIII cross-links fibrin, completing blood coagulation and protecting the hemostatic plug from the fibrinolytic activity at the clot formation site. In vitro studies demonstrated that supplementation with FXIIIC increases clot firmness assessed by TEM in perioperative patients with elevated FGN and reduced FXIII levels [31]. However, in another in vitro model of massive transfusion in trauma, combination therapies with FC and fresh frozen plasma, but not FXIIIC, improved both coagulation kinetics and fibrin-based clot strength [32, 33]. Our present study indicates that increasing concentrations of FXIII enhance clot strength at fixed concentrations of PC (1 IU/ml) and FGN (4 g/l). Consistently, there is further evidence that FXIII deficiency will impair FGN function and fibrin formation, suggesting an inverse link between low FXIII levels and enhanced thrombin generation, modifying the structure-function relationship of fibrin to support hemostasis [34]. Data derived from clinical studies propose maintenance of 50–60% of FXIII activity to avoid bleeding tendency in the perioperative setting [35].

CRF compositions without FXIII, yielding comparable clot strength in TEM when compared to our final composition, are possible from a theoretical point of view. We decided to add a purified source of FXIII to our final CRF composition despite the high potential of concentrate-derived FGN on viscoelastic clot strength to maintain a close-to-physiological factor composition.

The safe upper limit of FC treatment has not been precisely defined. It is currently suggested that plasma levels of FGN should reach 1.5 to 2 g/l in bleeding patients [36]. There is a clear tendency, as reported in different guidelines, to recommend elevating plasma FGN in some clinical situations [8, 37, 38]. Taking into consideration the results of our TEM studies it may be difficult to maintain a well-balanced coagulation factor composition during a long-lasting, high-dynamic bleeding event if supplements are only point-of-care driven and punctual. In this context, a fixed ratio of clotting factors in CRFs administered under volume therapy could provide more balanced stability within the complex multifactor system of blood coagulation than single factor substitutes as proposed in current algorithms.

CT in TEM is partially dependent on thrombin generation. Direct anticoagulants reducing thrombin generation definitively prolong CT [39]. Platelets contribute to enhance thrombin generation, accelerate CT, and increase MCF. Additionally, platelet phospholipids dramatically contribute to the amplification of coagulation mechanisms, thus potentiating thrombin generation and fibrin polymerization. Fibrin then interacts with activated platelets and plays a critical role in MCF. CT values of platelet-free CRF samples in our in-vitro experiments were significantly prolonged when compared to plasma CT levels of our internal control group. Several reasons may account for these findings:

First, our in vitro samples were completely free of any phospholipids or cell membrane fragments that could influence factor activation. Consequently, the addition of platelets to CRF containing 1 IU/ml PC, 4 g/l of FGN and 1 IU/ml FXIII leads to the normalization of CT and MCF. It could be assumed from our studies that, when combined with CRF, a platelet count around 100 × 10^3^/μl should be required to fully reconstitute TEM parameters to levels observed in whole blood studies (see Fig. 2a-c). CT values above 80 s are considered to reflect pathological thrombin generation and are generally accepted as treatment threshold. CT values of CRF combined with platelets were significantly shorter than this generally recommended treatment thresholds [15] (Tables 1 and 5).

Second, the used PCC in our experiments contains heparin. Other study groups previously reported about CT sensitivity of extrinsically activated TEM tests [40]. It is questionable if this phenomenon has any clinical relevance. The currently scientific rationale rather suggests that PCCs might be associated to overshot thrombin generation with the potential to induce disseminated intravascular coagulation and that Antithrombin III supplements might mitigate this potentially dangerous adverse effect [41]. The complete absence of antithrombin in the final CRF composition is a major limitation of our experiments and the effects of PCC supplements in clinical situations with reduced antithrombin levels will have to be analyzed in future trials.

A further limitation of our experimental studies is the complete absence of red blood cells. Red blood cells seem to exert a more important role in primary hemostasis, whereas their modulating effect on secondary hemostasis seems to be negligible [13]. The fact is that, viscoelastic studies can be reliably performed in plasma samples [12, 13]. Surprisingly, an inverse relation between hematocrit and clot firmness was previously reported under experimental and clinical anemic conditions [42]. We cannot rule out that the presence of red blood cells in our in vitro model could lead to a measurable reduction of clot firmness parameters. However, following reports of Schoergenhofer et al. [13] no effects on other TEM parameters should be expected under whole blood conditions. Under massive transfusion using CRF as a plasma substitute, transfusion of red blood cells would be an integral part of the clinical management to uphold an adequate amount of oxygen carriers within the circulating blood volume.

Altogether, the transfer of our data into a clinical context must therefore be done very carefully. All factor components of CRFs have previously been safely administered in loose compositions for the management of bleeding associated coagulopathy [43]. PCCs show a reliable safety profile and are now the treatment of choice for the emergency reversal of Vitamin K antagonists [44, 45]. Nevertheless, a careful assessment of the thrombogenic potential of fixed factor combinations for the treatment of a multiple factor deficit under massive bleeding will have to be performed in future studies.

## Conclusions

Coagulation factor concentrates suspended in albumin solutions have the potential to restore mechanisms of secondary hemostasis in the absence of any blood component, showing viscoelastic properties comparable to whole blood when tested in presence of platelets. Coagulation factor enriched albumin-based colloids could be a valuable tool to provide stable intravascular volume effects in hypovolemic conditions and to simultaneously maintain basic coagulation mechanisms. This could offer future alternatives to transfusion of fresh frozen plasma under resuscitation conditions.

## Data Availability

The dataset used and/or analysed during the current study are available from the corresponding author on reasonable request.

## Abbreviations

AFS: Artificial Fluid Solution
A10: Amplitude at 10 min
CFC: Coagulation Factor Concentrate
CRF: Coagulation Resuscitating Fluid
CT: Clotting Time
FC: Fibrinogen Concentrate
FGN: Fibrinogen
FXIII: Factor XIII
FXIIIC: Factor XIII Concentrate
MCF: Maximum Clot Firmness
PC: Prothrombin Complex
PCC: Prothrombin Complex Concentrate
TEM: Thromboelastometry
